# Psychometric properties and norm values of a short screening version of the profile of mood states POMS from the German general population

**DOI:** 10.1038/s41598-025-07388-6

**Published:** 2025-07-04

**Authors:** Katja Petrowski, Monika Bjelopavlovic, Markus Zenger, Elmar Brähler, Bjarne Schmalbach

**Affiliations:** 1 Medical Psychology and Medical Sociology, University Medical Center of the Johannes Gutenberg-University, Mainz, Germany; 2 Department of Internal Medicine III, University Medical Center Carl Gustav Carus at the University of Dresden, Dresden, Germany; 3 Department of Prosthetic Dentistry, University Medical Center of the Johannes Gutenberg-University, Mainz, Germany; 4 Faculty of Applied Human Studies, University of Applied Sciences Magdeburg and Stendal, Stendal, Germany; 5 Integrated Research and Treatment Center (IFB) AdiposityDiseases-Behavioral Medicine, Psychosomatic Medicine and Psychotherapy, University of Leipzig Medical Center, Leipzig, Germany; 6 Department of Psychosomatic Medicine and Psychotherapy, University Medical Center of the Johannes Gutenberg-University, Mainz, Germany

**Keywords:** Mood, Assessment, Scale development, Screening instrument, Factor analysis

## Abstract

The current study aimed to provide further evidence of the structural validity of the 16-item-short version of the Profile of Mood States (POMS), a widely-used tool for assessing an individual’s emotional state. This is significant for various research inquiries in clinical and social psychology. In order to cross-validate previous findings, an additional evaluation of the factorial structure and the psychometric properties is necessary in a newly collected dataset. A representative sample for age and gender of *N* = 2503 with 1329 (53%) female, 1173 (47%) male, and 1 (< 1%) diverse, with a mean age of *M* = 46 (*SD* = 18) was collected. The model fit for the four-factor model was acceptable, with good reliability for all factors. We found evidence for (partial) strict invariance between gender and age groups. There were small to moderate group differences for the Anger and Vigor subscales regarding age. We report normative percentile ranks. Our findings suggest that the POMS-16 is a dependable and structurally valid gauge of mood states. Especially in situations where a brief and cost-effective assessment is preferred, the POMS-16 should be a considered option.

## Introduction

The Profile of Mood States questionnaire (POMS) is a widely used questionnaire to assesses the mental state of various medical patients, including those with heart surgery, cataracts, epilepsy, and sleep apnea syndrome^1–4^. POMS has also been used in (psycho)oncology to evaluate various outcomes, such as quality of life, stress levels or the impact of interventions^5^. More frequently, POMS measures psychological and pharmacological quantities in clinical, as well as in occupational and sports medicine studies^6–8^.

The original American version of POMS^9,10^ consists of six mood dimensions, assessed with 65 items rated on a 5-point scale. The mood dimensions Anger-Hostility, Confusion-Bewilderment, Depression, Fatigue-Inertia, and Tension-Anxiety are summed up and Vigor-Activity is subtracted from the sum to obtain the Total Mood Disturbance Scale. Internal consistency coefficients range from 0.90 to 0.94, and retest reliabilities range from 0.65 to 0.74 in clinical samples^10^. Similar values were reported non-clinical samples, with higher retest reliabilities over a one-week interval^11^.

In other versions POMS utilizes two main prompts on a 7-step answer scale: 'How have you felt during the past week including today?' and 'How do you feel right now?' The ‘past week’ framing is usually preferred to the ‘right now’ framing as it measures reoccurring states and at the same time retains sensitivity to interventions. And although McNair et al.^10^ replicated the original structure using the ‘right now’ framing, it should be evident that perceptions of intensity, seriousness, and frequency of episodes may vary with the reference period. In Anglo-Saxon contexts, scores from the ‘past week’ instruction were higher than those from multiple ‘right now’ assessments. Terry et al.^12^ recommended using the ‘right now’ prompt due to recall being influenced by mood and significant events.

Although the internal structure of the Profile of Mood States (POMS) is well-validated, the Confusion factor is regarded as a cognitive state. Some adaptations have retained the Friendliness component due to the restricted scope of pleasant mood states encompassed^13,14^.

Other factors that can affect mood state responses, such as diverse mood state descriptors, response formats, and assessment circumstances have been discussed in previous research. Especially, the circumstances of mood state assessment, including the timing and location, are crucial measurement elements^12,15,16^. Overall, instruction type and test administration conditions both play important roles and the invariance of mood scores needs to be ascertained in the interpretation of mood states^12,16,17^.

The 35-item version of the POMS (German version by Biehl et al.^18^) generally appears to be the most widely used and it consists of the four scales: dejection/anxiety, fatigue, vigor, and anger, evaluated using a 5- or 7-point response scale. Satisfactory psychometric properties were reported for this version based on a student sample. However, when applied to a general population sample, only a limited satisfactory factorial structure was observed^19^. Therefore, Petrowski et al.^20^ conducted an item selection for a robust factorial structure. Using exploratory factor analysis and model comparisons of potential item subsets^21^, a four dimensions scale with total of 16-item set was identified, ensuring good reliability and factorial structure. Confirmatory factor analysis showed a good fit and high reliability for the subscales (0.86 to 0.91). This 16-item short version is strictly invariant across age groups, with strong and partial strict invariance by sex.

This 16-item short version was developed from an older dataset of the long version. To ensure its validity and independence from the excluded 19 items of the POMS-35, further evaluation of the factorial structure and psychometric properties is necessary using a newly collected dataset exclusively implementing the 16-item version. Thus, the current study aims to evaluate the factorial structure and psychometric properties of the 16-item short version with up-to-date norm values from a representative sample of the German general population.

## Method

The present investigation was part of a representative survey of the German general population. An independent institute for opinion and social research (USUMA, Berlin, Germany) organized and carried out the data collection. Participants were required to be at least 14 years of age and sufficient German-speaking capabilities. In addition to providing socio-demographic information, participants completed several self-report questionnaires on physical and psychological symptoms. The study participants were selected by means of a random-route sampling method with 258 sample points. Initially, 5418 households were selected, and 5389 were deemed eligible for participation. The Kish selection grid^22^ was then utilized to select individuals within households. In total, 2503 individuals took part in the survey (46% of those contacted). The study protocol was approved by the ethics committee of the University of Leipzig (043/20-ek) and adhered to ICH-GCP guidelines, the ICC/ESOMAR International Code of Marketing and Social Research Practice, and the Declaration of Helsinki. After being educated about the study procedures, data collection, and anonymization of personal data, all participants gave verbal informed consent, in accordance with German law.

### Instruments

In the present study the short version of the Profile of Mood States (POMS^23^) by Petrowski et al.^20^ was implemented. The short version consists of 16 items spread across 4 scales: dejection / anxiety (4 items), fatigue (4 items), vigor (4 items) and anger (4 items). Similar to the different English versions the 16 items were answered on a 7-point Likert scale and evaluated for "the last 24 h".

### Statistical analysis

All analyses were performed in R using the packages *dynamic*, *lavaan*, and *semTools*^24–26^. The analysis script is published at https://github.com/bschmalbach/POMS_Validation/blob/main/POMS16.Rmd. For the confirmatory factor analysis, we utilized robust full-information maximum likelihood estimation (FIML). We made this decision because there were a sizable number of respondents with missing values (mostly singular values): 143 (5.7%) individuals had at least one missing value. Thus, by using FIML, we were able to use all available information and not selectively include responses based on missingness. Furthermore, the items exhibited only a slightly positive skewness of 0.62, and thus robust maximum likelihood estimation appears acceptable. To assess the model fit, we considered the Comparative Fit Index (CFI), the Tucker-Lewis Index (TLI), the Root Mean Squared Error of Approximation (RMSEA), and the Standardized Root Mean Squared Residual (SRMR). We employed cutoffs of 0.95 for CFI and TLI, and 0.08 for RMSEA and SRMR^27,28^. To supplement, this analysis we also considered the dynamic cutoff values as proposed by McNeish and Wolf^29^. To evaluate scale reliability, we examined McDonald’s ω^30^.

To analyze differences between sociodemographic groups, we first tested for measurement invariance by comparing increasingly restrictive models: configural invariance (baseline model), metric invariance (equal loadings across groups), scalar invariance (equal loadings and indicator intercepts across groups), and strict invariance (equal loadings, indicator intercepts, and indicator residuals across groups). The fit should not decrease by more than 0.01 in terms of CFI and RMSEA between models^31,32^. To supplement these analyses, we report RMSEA_D_ according to Savalei and colleagues^33^. Its interpretation is equivalent to the standard RMSEA index with the customary 0.08 cutoff. For the ANOVA comparisons and normative data, only respondents with complete data on a given subscale were included in the analysis. We conducted ANOVAs to check whether there are meaningful differences in POMS scores between sociodemographic groups. This served the primary purpose of determining the necessity of dedicated norm values for each subgroup. In addition to conducting the ANOVAs, we checked for variance homogeneity and normality of residuals—both assumptions were fulfilled or only violated mildly. The normative values were then computed based on percentile ranks for each given sum score.

## Results

### Sample

The initial study sample consisted of 2503 responsdents. Of those, 1329 (53%) were female, 1173 (47%) were male, and 1 was diverse (< 1%). The mean age was 46 (*SD* = 18), which we split into three even age groups: ≤ 36 years (*n* = 824, 33%), 37–55 years (*n* = 808, 32%), and > 55 years (*n* = 871, 35%). A more detailed description is reported in Table 1.

**Table 1:** Sample description.

	n	%
Sex	2503	
Male	1173	46.9
Female	1329	53.1
Diverse	1	0
Age groups	2503	
Young (1st tertile)	824	32.9
Middle-aged (2nd tertile)	808	32.3
Old (3rd tertile)	871	34.8
Relationship status	2487	
Married, living together	981	39.4
Married, separated	69	2.8
Unmarried	992	39.9
Divorced	296	11.9
Widowed	149	6
Education	2496	
Not graduated (yet)	136	5.4
Less than 10 years	507	20.3
10 years	1090	43.6
More than 10 years	763	30.6
Net household income per month, in €	2319	
Up to 1,000	194	8.4
Up to 1,500	328	14.2
Up to 2,500	585	25.3
Up to 3,500	536	23.1
Up to 5,000	453	19.5
More than 5,000	223	9.6

### CFA

The 4-factor model established by Petrowski et al. (2021) exhibited acceptable fit in this sample when compared to the customary fixed cutoffs, χ^2^(98) = 819.66, *p* < 0.001, *CFI* = 0.957, *TLI* = 0.947, *RMSEA* = 0.056, *SRMR* = 0.040. Only the *TLI* slightly falls below the threshold of acceptability. The dynamic cutoffs largely replicate these findings: Level 1 misspecified models would yield *SRMR* = 0.070, *RMSEA* = 0.047, and *CFI* = 0.977, Level 2 models would yield *SRMR* = 0.077, *RMSEA* = 0.068, and *CFI* = 0.959, and Level 3 misspecification would yield *SRMR* = 0.084, *RMSEA* = 0.088, and *CFI* = 0.940. This puts our empirically determined fit values pretty convincingly into Level 2 (with SRMR being better than expected). This corresponds to “fair” fit according to McNeish and Wolf. Reliability (ω) of the factors was good, ranging between 0.859 and 889.

Additionally, we analyzed a unifactorial model—since the POMS is often summarized using a Total Mood Disturbance Score. Our findings show that such a model is completely unacceptable, χ^2^(104) = 6339.45, *p* < 0.001, *CFI* = 0.621, *TLI* = 0.563, *RMSEA* = 0.155, *SRMR* = 0.126, ω = 0.716.

### Measurement invariance

Table 2 shows the results of the measurement invariance tests. For gender, there were no meaningful differences in the measurement model at any level. That is, strict invariance can be assumed. In contrast, age groups exhibited some differences with regard to the residuals. Thus, we can only assume partial strict invariance. Specifically, we freed the residuals of the two items with the largest deviations between the age groups: “full of pep” and “vigorous”. This indicates that while the vigor factor may be comparable across ages (given metric and scalar invariance), the error terms aren’t—which is an indication for differing reliability between groups.

**Table 2:** Tests of measurement invariance.

	χ^2^	Δχ^2^	*df*	Δ*df*	*p*	*CFI*	Δ*CFI*	*RMSEA*	Δ*RMSEA*	RMSEA_D_
Gender invariance										
Baseline model	914.50		196			.957		.056		
Metric invariance	921.75	7.25	208	12	.841	.958	.001	.054	.002	.000
Scalar invariance	971.89	50.14	220	12	< .001	.956	.002	.054	.000	.036
Strict invariance	989.67	17.78	236	16	.337	.954	.002	.052	.002	.007
Age invariance										
Baseline model	1029.07		294			.958		.056		
Metric invariance	1067.01	37.91	318	24	.035	.958	.000	.055	.001	.015
Scalar invariance	1260.18	193.17	342	24	< .001	.949	.009	.058	.003	.053
Strict invariance	1524.03	263.85	374	32	< .001	.933	.016	.063	.005	.054
Partial strict invariance^a^	1415.24	155.06	370	28	< .001	.939	.010	.060	.002	.043

^a^The residuals of items 15 and 16 were allowed to vary between groups for this model.

### Group differences and norm values

As can be seen in Table 3, there were small-to-moderate differences between age groups with regard to the Anger and Vigor subscales. Specifically, younger respondents reported higher values in both subscales. Some significant yet smaller (*R*^2^ < 0.01) differences were observable for other comparisons as well. Even though these differences are relatively small, they demonstrate the need for dedicated normative values for each subgroup. Tables 4, 5, 6, 7 display normative values for the German general population.

**Table 3:** Group comparisons.

	*F*	*p*	*R*^2^
Anger			
Gender	0.24	.627	< .001
Age groups	25.08	< .001	.020
Fatigue			
Gender	19.97	< .001	.008
Age groups	7.45	< .001	.006
Vigor			
Gender	2.36	.125	< .001
Age groups	19.17	< .001	.016
Dejection			
Gender	13.16	< .001	.005
Age groups	5.80	.003	.005

**Table 4:** Normative percentile ranks, anger subscale.

	Male			Female		
	≤ 36	37–55	> 55	≤ 36	37–55	> 55
4	19.1	27.5	20.3	22	26.6	16.3
5	28.2	37.1	28.9	31.9	37.8	24.2
6	37.8	45.2	34.7	38.8	47.8	31.7
7	45.3	55.1	43.3	45.4	56.6	40.3
8	51.9	62.9	49.1	54.6	63.3	45.6
9	61.6	68.4	53.7	59.3	70.5	51.8
10	67.4	72.5	59.5	66.4	74.2	58.5
11	70.7	76.5	66.3	70.4	81	63.8
12	75.1	80.8	71.9	73.8	83.8	68.6
13	80.9	83.3	76.7	76.8	86.7	73.9
14	84	86.9	81.3	81.8	88.4	78.2
15	85.9	88.9	84.8	83.7	90	81.5
16	89.8	92.7	87.3	89.1	93	85.9
17	91.2	94.9	90.4	91	95	87.5
18	92	96.7	92.4	93.1	95.4	90.6
19	93.9	97	93.9	94.3	95.9	92.6
20	96.1	98	94.9	96.5	96.9	93.8
21	96.7	99	95.7	97.2	97.2	94.7
22	98.1	99.7	97	97.9	98	95.9
23	98.6	100	98.2	98.1	98.7	97.1
24	98.9	27.5	98.7	98.8	99.1	97.4
25	99.7	37.1	99.2	99.3	99.3	98.3
26	100	45.2	99.7	100	99.8	98.6
27	19.1	55.1	100	22	100	99.3
28	28.2	62.9	20.3	31.9	26.6	100

**Table 5:** Normative percentile ranks, fatigue subscale.

	Male			Female		
	≤ 36	37–55	> 55	≤ 36	37–55	> 55
4	7.2	11.4	12.4	7.2	10.9	8.5
5	13.8	17.9	16.8	11.2	15.3	13
6	19.6	23.2	23.2	16.1	22.3	16.9
7	27.9	32.8	30.9	22.4	30.1	24.2
8	35.6	40.7	38.1	28	36	30.2
9	40.3	47.5	45.4	33.1	40	36.5
10	47.5	52	51	41	48	44
11	52.8	57.6	55.7	48.3	54.8	50
12	57.7	64.6	62.9	53.8	59.6	56.3
13	63.3	71	68.3	59	63.5	58.9
14	70.4	76.5	73.5	63.6	69.9	63
15	75.1	80.1	77.6	67.6	74.5	68.6
16	80.1	85.6	82.2	74.6	79.9	74.9
17	84	88.1	86.3	78.1	83.4	78.3
18	86.7	91.2	89.9	81.8	88.4	82.6
19	89.5	92.7	93	84.4	91.3	86.2
20	92.5	94.7	94.1	88.3	93	88.6
21	94.2	95.7	95.9	89.5	94.8	90.8
22	94.5	96	97.7	91.4	96.1	92.3
23	95.3	97	98.7	92.8	96.7	94.4
24	96.4	98.5	99	94.4	98	96.1
25	97.8	99.2	99.2	97	98.5	97.3
26	98.9	100	99.5	97.9	98.9	98.3
27	99.2	11.4	99.7	98.6	99.3	98.8
28	100	17.9	100	100	100	100

**Table 6:** Normative percentile ranks, vigor subscale.

	Male			Female		
	≤ 36	37–55	> 55	≤ 36	37–55	> 55
4	1.4	1.8	1.3	1.4	1.8	0.2
5	1.7	2.5	1.5	1.6	3.3	0.7
6	3	3.8	2.6	2.1	4.5	1.2
7	3.9	4.3	2.8	3.1	6.2	1.9
8	4.7	6.6	3.3	4.2	8.5	2.9
9	6.9	7.9	4.9	6.6	10.9	4.1
10	9.1	8.9	6.6	8.9	14	6.8
11	11.3	11.7	9.2	11.5	17.4	9.7
12	15.2	15.3	12.5	15	22.7	13.6
13	19.6	22.1	17.4	19.2	28.7	16.9
14	24.6	27.5	23	24.6	35.4	20.8
15	29.3	32.3	27.6	32.2	40.1	27.8
16	36.5	41.2	34.5	39.4	47.2	38.5
17	43.6	50.9	39.6	44.6	55.2	44.8
18	50	57.3	50.1	53.1	62.6	52.5
19	62.2	66.9	58.6	62	69.9	60
20	73.8	78.9	68	72.8	79.3	70.2
21	79	86.5	73.7	77.2	85.7	75.8
22	84.8	90.3	80.1	82.2	89.5	82.6
23	88.4	93.1	84.1	86.9	93.1	86.9
24	95.9	96.9	90.8	94.8	96.9	92
25	97.5	98.5	94.4	97.7	98.2	95.4
26	97.8	99.2	97.4	98.6	98.4	98.1
27	99.2	99.7	98.5	99.3	99.3	99
28	100	100	100	100	100	100

**Table 7:** Normative percentile ranks, dejection subscale.

	Male			Female		
	≤ 36	37–55	> 55	≤ 36	37–55	> 55
4	34.1	35	32.6	27.8	32.4	23.3
5	47.1	48.5	41.9	39.8	44.6	34.1
6	56.8	54.8	49.4	48.6	53.3	43.8
7	61.8	63.5	57.6	56.7	60	52.9
8	68.7	70.8	65.1	63.2	66.5	59.4
9	74.8	74.2	69.3	69	71.3	64.4
10	78.7	78.2	73.6	73.8	75.2	70.4
11	83.7	82.2	78.6	77.3	79.8	74.5
12	84.5	84.8	84.2	81	83	78.6
13	87.3	87.5	87.1	84.7	85.7	82.7
14	89.8	89.8	88.6	88.7	88	84.6
15	92	92.8	91.2	89.8	90.2	86.3
16	93.4	94.8	93.5	91.7	93.7	88.9
17	95.3	96.8	95.1	94.2	95	91.3
18	97	98.2	96.9	95.4	95.9	92.8
19	97.5	98.8	97.2	96.5	97	93.8
20	97.8	99.5	98.7	97	97.8	94.5
21	98.6	99.8	99.2	97.7	98.5	95.2
22	99.2	100	99.5	98.1	98.7	95.7
23	99.4	35	100	98.8	98.9	96.9
24	99.7	48.5	32.6	99.8	99.3	98.3
25	100	54.8	41.9	100	99.6	98.8
26	34.1	63.5	49.4	27.8	100	99.3
27	47.1	70.8	57.6	39.8	32.4	99.5
28	56.8	74.2	65.1	48.6	44.6	100

## Discussion

The "Profile of Mood States" (POMS^10^) is a widely used questionnaire in clinical research. For epidemiological studies, short instruments with strong psychometric properties are essential. Therefore, a short screening version was derived from the long form. To cross-validate those results in a sample featuring only the final 16 items, a representative sample of the general population in Germany was collected. The study aimed to evaluate the psychometric properties, factorial structure, and norm values of the 16-item short version of the POMS.

Based on the newly collected representative dataset, the model fit for the four-factor model was acceptable, with good reliability for all factors. We found evidence for (partial) strict invariance across gender and age groups. Small to moderate differences were observed for the Anger and Vigor subscales regarding age. Further, results from the unifactorial CFA discourage from the usage of the Total Mood Disturbance Score. The English version of the POMS by Cella et al.^34^ consists also of a small number of items (11 items) but only provides a Total Mood Disturbance score without subscales. Therefore, the 16-item version provided here is the shortest available version of the POMS that maintains subscale measurements. Furthermore, it previously showed a high correlation with the long 35-item version^20^.

While the large sample size and broad age range are strengths of this study, the representativeness for the general population may limit applicability to samples with altered moods, such as clinical settings. To further validate or refute the factorial structure, the POMS should be applied to diverse groups, including clinical samples. In addition, further consideration should be given to the process of external validation, specifically the usage of concrete criteria (such as behaviors) which the POMS could predict.

In sum, this investigation presents evidence of the POMS-16’s structural validity and reliability. It can be recommended for social, personality, and clinical research interested in changes in affect and mood.

## Data Availability

Datasets are available upon reasonable request from Katja Petrowski at kpetrows@uni-mainz.de.
